# Closed-Loop Bowel Obstruction Induced by Ventriculoperitoneal Shunt Catheter Coiling at the Sigmoid Colon: A Case Report

**DOI:** 10.7759/cureus.49045

**Published:** 2023-11-19

**Authors:** Rinad M AlJoaid, Hawra H Alshakhori, Arwaa Haji, Dunya Alfaraj, Murad F Alabbad

**Affiliations:** 1 Emergency Department, Imam Abdulrahman bin Faisal University, King Fahd University Hospital, Dammam, SAU

**Keywords:** vps coiling, adult, complication, intestinal obstruction, ventriculoperitoneal (vp) shunt

## Abstract

Intestinal obstruction is a rarely encountered complication in patients with ventriculoperitoneal (VP) shunt. The most common causes of bowel obstruction in this subset of patients include volvulus, formation of a spontaneous knot, and adhesions. Herein, we report a 21-year-old bedridden male with a history of congenital hydrocephalus on VP shunt, spina bifida, neurogenic bladder, and paraplegia who presented with a seven-day history of abdominal discomfort, distention, constipation, vomiting, and intolerance to oral intake. Abdominal x-ray showed dilated bowel loops. Computed tomography (CT) of the abdomen demonstrated a closed-loop bowel obstruction at the level of the sigmoid colon caused by the coiling of the VP shunt catheter. Diagnostic laparoscopy revealed the VP shunt tube coiling around a segment of the sigmoid colon with no signs of bands, ischemia, or perforation. Pulling and shortening of the tube was done. The procedure went uneventfully, and the patient was discharged home in stable condition. Maintaining a high index of suspicion for knotting the peritoneal catheter around the bowel is crucial when a patient on a VP shunt presents with a picture suggestive of intestinal obstruction. Early surgical intervention might be required to prevent further progression and complications.

## Introduction

A ventriculoperitoneal shunt (VP) is a neurosurgical procedure that involves the insertion of a tube into the brain's ventricles to drain excess cerebrospinal fluid (CSF), which relieves pressure on the brain [1]. The procedure treats conditions such as hydrocephalus, in which there is an excessive accumulation of CSF in the brain [1]. Other common indications for VP shunt include intracranial hemorrhage, brain tumours, spina bifida, and cerebral edema [1].

The placement of a VP shunt is associated with several complications [2]. The rate of complications remains substantially high, with around 40% of shunt failure cases occurring within the first year of shunt implantation [3,4].

The complications could be mechanical or non-mechanical [5]. Non-mechanical complications include infection of the shunt tract, meningitis, peritonitis, pseudocyst formation, cerebrospinal fluid leakage, pleural effusion, and ascites [5,6]. Mechanical complications include failure of the proximal or distal catheter of the shunt, which can be attributed to disconnection, obstruction, or migration [5,6].

The most common cause of shunt malfunction is shunt obstruction, which could happen in the proximal or distal catheter, with the first being the most common [3]. The second most common cause of shunt malfunction is infection [3].

Abdominal complications following VP shunt insertion include catheter migration through the GI tract, umbilicus, vagina, and scrotum, formation of peritoneal pseudocyst, pseudotumor of the mesentery, peritonitis, ascites, volvulus, intestinal obstruction, and bowel perforation [7,8].

Intestinal obstruction is a rarely encountered complication in patients with a ventriculoperitoneal shunt [9]. The most common causes of bowel obstruction in this subset of patients include volvulus, formation of a spontaneous knot, and adhesions [9,10]. In patients on a VP shunt presenting with a picture suggestive of intestinal obstruction, including abdominal pain, distention, vomiting, and constipation, prompt recognition and management are crucial in order to prevent subsequent ischemia, necrosis, and perforation of the bowel [2,9,10].

## Case presentation

A 21-year-old bedridden male with a known medical history of congenital hydrocephalus on a VP shunt, spina bifida, neurogenic bladder, and paraplegia presented with a seven-day history of diffuse, intermittent abdominal discomfort. The patient had been on a cecostomy tube for 15 years and was recently diagnosed with intestinal amebiasis. He reported subjective abdominal distention, absolute constipation, passage of clear rectal discharge without blood, vomiting of gastric juice, and intolerance to oral feeding. The patient had no known allergies.

Upon physical examination, the patient appeared calm and was not in distress. The abdomen was distended without palpable masses, and mild left flank tenderness and normal bowel sounds were noted. There were no signs of peritonism, and the hernial orifice was intact. A digital rectal examination revealed an empty rectum with clear discharge and no evidence of blood.

A complete blood count (CBC) was obtained at admission and showed evidence of leukocytosis, thrombocytosis, neutrophilia, and lymphocytopenia. Liver function tests (LFT) showed elevated total bilirubin, direct bilirubin, total protein, and lactate dehydrogenase. Prothrombin time was prolonged. Urine analysis showed evidence of hematuria and ketonuria. Venous blood gas showed low pH, partial pressure of carbon dioxide (PCO2), partial pressure of oxygen (PO2), potassium, and bicarbonate.

**Figure 1:**
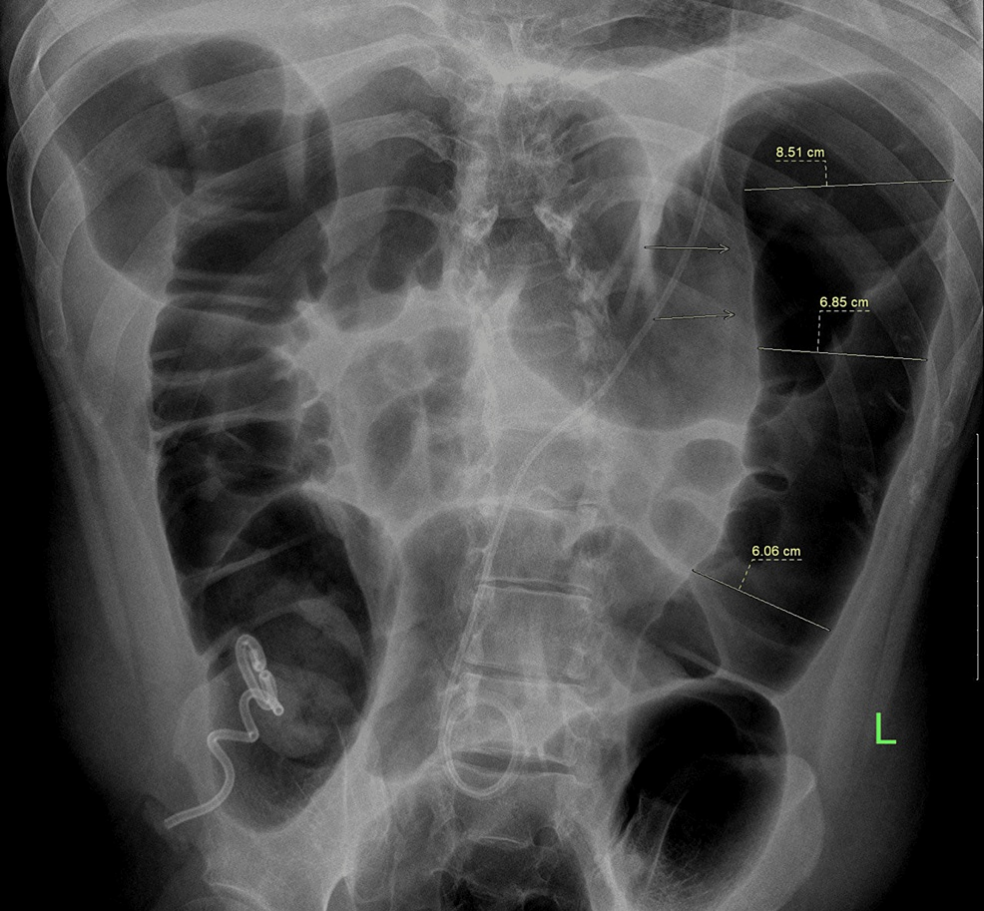
X-ray abdomen showing dilated large bowel loops

Abdominal X-ray showed evidence of dilated large bowel loops (Figure 1).

**Figure 2:**
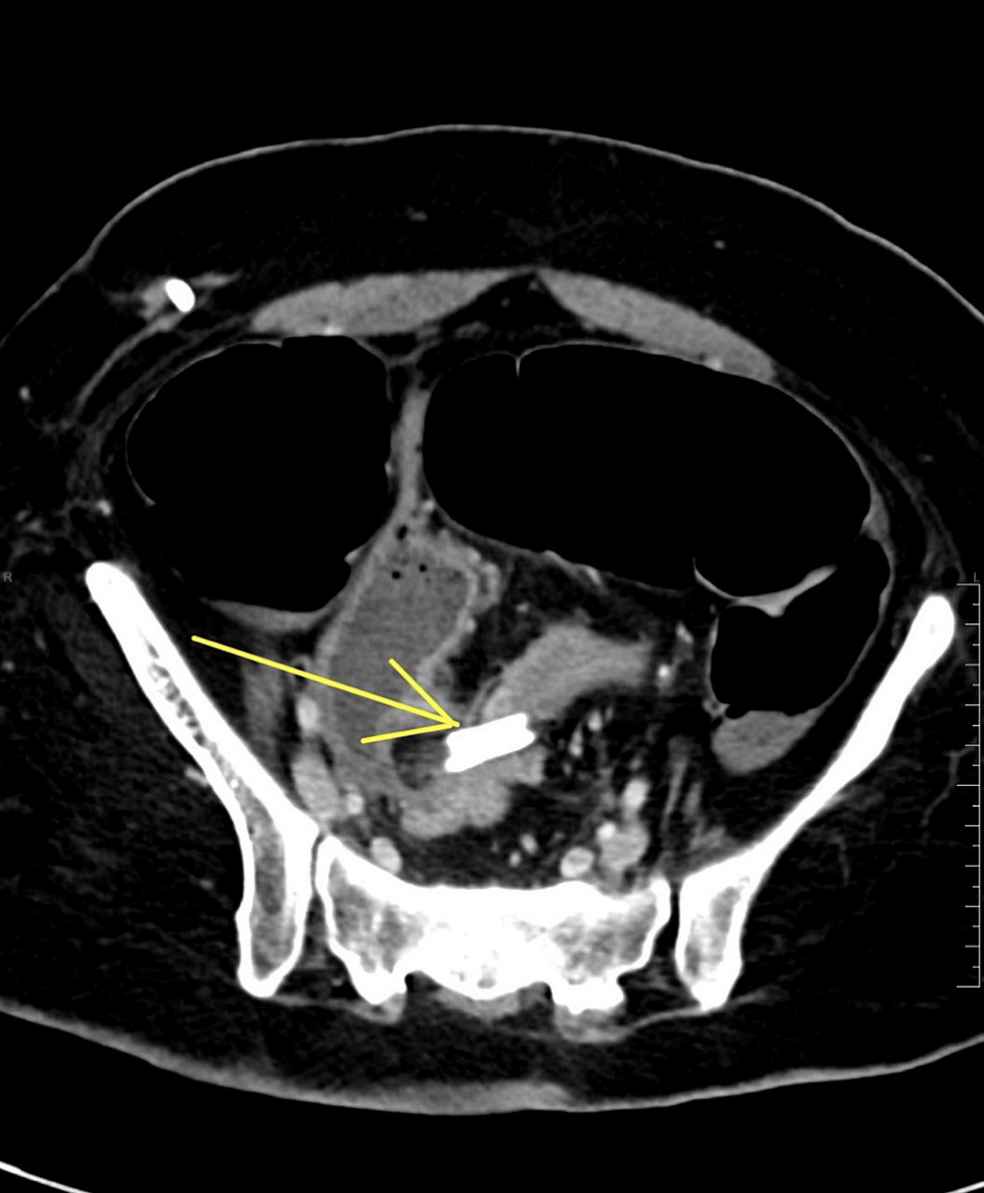
Computed tomography of the abdomen showing a closed-loop bowel obstruction at the level of the sigmoid colon caused by the coiling of the ventriculoperitoneal shunt catheter

Computed tomography (CT) imaging of the patient's abdomen demonstrated a closed-loop bowel obstruction at the level of the sigmoid colon caused by the coiling of the VP shunt catheter with proximal dilatation of the large bowel. Within the closed-loop bowel obstruction, there were two segments of significant bowel wall thickening, containing fluid with subtle enhancement adjacent to the coiled lower VP shunt tube; free fluid was also noted in the pelvic region, concerning for ischemic changes. There was no free air seen in the abdomen or pelvis (Figure 2).

The patient was admitted to the regular ward under the care of the general surgery department as a case of sub-acute intestinal obstruction secondary to coiling of the VP shunt tube. An order of "nothing per mouth" was placed, and a nasogastric tube (NGT) and Foley catheter were inserted. The patient received intravenous fluids, antibiotics, analgesia, pantoprazole, and metoclopramide.

On the fourth day of admission, the patient underwent a diagnostic laparoscopy. Exploration of the abdomen revealed that the VP shunt tube was coiled around a segment of the sigmoid colon. The tube was pulled without resistance and shortened by 33 cm as recommended by the neurosurgery team, who were consulted intra-operatively. The bowel appeared healthy with no signs of bands, ischemia, or perforation. Free serous fluid was noted in the pelvis and was aspirated. The procedure went smoothly without any complications.

During the postoperative period, the patient showed clinical improvement. Oral feeding started and advanced gradually, and the patient tolerated it well. On the second postoperative day, the patient passed flatus and stool. The patient was discharged in a stable condition two days after the surgery.

## Discussion

VP shunt placement is the established procedure for treating infant hydrocephalus [11]. However, this procedure is associated with several complications [2]. Complications related to the abdominal area have been reported in 25-30% of patients who undergo this procedure [6]. One of the exceedingly rare complications of VP shunt insertion is the formation of knots on the peritoneal catheter [12]. Intestinal obstruction and subsequent necrosis due to coiling or knotting of the peritoneal catheter are also highly uncommon [13]. 

The mechanism by which a VP shunt's peritoneal catheter coiling or knotting occurs has yet to be fully understood [14]. However, several predisposing factors have been suggested, including increased peristalsis, a crowded abdominal cavity, and intra-abdominal adhesions [14]. In addition, it has been suggested that a catheter of higher elasticity, greater length, and smaller diameter can contribute to the formation of a knot [14].

The time interval between shunt placement and the onset of intestinal obstruction symptoms is very variable [13]. Mechanical obstruction is often encountered when removing the catheter's abdominal end [13]. It is possible that when looping of the excessive length of the abdominal catheter occurs around a part of the bowel, trials to retrieve the catheter can tighten the loop, resulting in coiling or knotting around the bowel, which can result in bowel strangulation and necrosis [15]. Before shunt revision surgeries, reviewing pre-operative shunt series can help identify any existing loops or knots of the peritoneal catheter [15]. Only one case described a VP shunt inserted in a pediatric patient, with multiple shunt revisions for recurrent hydrocephalus, where a knot formation manifested in adulthood with small intestinal obstruction [16]. Another case reported bowel obstruction caused by knotting of the VP shunt in an adult patient who had the shunt placement during infancy with no subsequent manipulation [17].

Intestinal obstruction secondary to knot formation or coiling of the peritoneal catheter of the VP shunt carries a risk of bowel necrosis and perforation [15]. During shunt revision surgeries, when trials to remove the peritoneal catheter are met with resistance, excessive pulling should be avoided as it can strangulate the bowel and result in bowel necrosis [15]. A case report described a three-month-old girl with a history of myelomeningocele and Chiari II malformation with VP shunt insertion at birth who underwent shunt removal and insertion of an external ventricular drain due to shunt infection [15]. During the surgery, trials of pulling out the peritoneal catheter failed due to resistance, which was initially thought to be caused by trapping the catheter by adhesions [15]. Therefore, the catheter was cut with the distal end remaining in the abdomen [15]. Post-operative imaging revealed a formation of a knot in the right lower quadrant of the abdomen, and it was planned to remove it during the placement of the new VP shunt as the patient was doing well. However, during the post-operative period, the patient developed small bowel obstruction due to coiling of the distal end of the catheter around a segment of the bowel, which was further complicated by bowel ischemia and necrosis that necessitated bowel resection [15].

It is crucial to have a high index of suspicion in patients with VP shunt. Knotting of the peritoneal catheter around the bowel should be considered when the patient presents with signs and symptoms of intestinal obstruction [2]. Early surgical intervention might be warranted to prevent further progression and complications [17].

## Conclusions

This case report clearly states that suspicion should always be maintained when a patient presents, regardless of how soon after insertion of prosthetic material in the body. Although the incidence of such complications is extremely low, their seriousness cannot be underestimated, and all neurosurgeons must be aware of the potential complications associated with VP shunts in adults. Recognizing these complications can be challenging due to their rarity, but neurosurgeons must maintain a heightened awareness and perception of VP shunt-related complications in adult patients.
